# Prevalence of pulmonary disease in alcohol and poly substance abuse

**DOI:** 10.1192/bjo.2021.590

**Published:** 2021-06-18

**Authors:** Ghazanfar Sheikh

**Affiliations:** CGL

## Abstract

**Aims:**

The aim of this study is to identify the prevalence of pulmonary disease or progression of symptoms which can lead to respiratory disease in substance misuse. Identify risk: prescribe a safer substitute treatment (Methadone vs Buprenorphine). Identify combination of different drugs, causing toxicity and damage to lungs.

**Background:**

Smoking and illicit substance misuse is recognized as a cause of pulmonary disease

**Method:**

The study was conducted in 2015 at outpatient addictions service (CAPS/CDC 69 Warwick Road London SW5 9HB). All 50-participants gave consent and data were collected including demographic details, history of substance abuse, route of administration, medical history, Peak Expiratory Flow rate (PEFR). A questionnaire was designed to assess the severity of shortness of breath (SOB), with or without exertion to ascertain the extent of the disease.

**Conclusion:**

14 males and 3 females achieved 31 to 48.9 % of predicted PEFR. 13 males and 7 females achieved 51.1 to 69.6% of predicted PEFR. 7 males and 2 females achieved 70.8 to 91.1% of predicted PEFR. 2 males and 2 females achieved 100 to 114.3% of predicted PEFR. From the questionnaire on SOB with or without exertion, 8 males and 2 females reported moderate to severe shortness of breath on minimum exertion. 33 males and 11 females reported variable degree of SOB. 4 males and 2 females did not report SOB on reasonable exertion. 23 males and 10 females reported variable degree of SOB at rest, 14 males and 3 females reported no SOB at rest. 34 participants were receiving OST with methadone, 16 were on Buprenorphine. 6 patient treatments were switched to Buprenorphine due to Exacerbated COPD. 47 patients reported using Tobacco from harmful to dependent use. 44 patients reported harmful to dependent use of crack cocaine. 34 patients fulfilled the criteria of harmful use of cannabinoids. 33 patients were diagnosed with harmful to dependent use of alcohol. 10 patients reported heavy use of solvents. 2 patients reported harmful use of Party drugs. Many patients reported a history of recurrent chest infections 3-6 times a year, Asthma, COPD, Emphysema, Pneumonia and 2 were treated for Tuberculosis. 1/3 patients have a diagnosis of hypertension and IHD, liver disease and portal hypertension

